# Surgical Management of an Isolated Posterior Manubrium Fracture in a Division One Collegiate Ice Hockey Athlete: A Case Report

**DOI:** 10.7759/cureus.92145

**Published:** 2025-09-12

**Authors:** Andin E Fosam, Jacob Friedman, Connor Mitrovich, Justin Blasberg, Christina R Allen

**Affiliations:** 1 Department of Orthopedic Surgery, Yale University, New Haven, USA; 2 Department of Yale Athletic Medicine, Yale University, New Haven, USA; 3 Department of Thoracic Surgery, Yale University, New Haven, USA

**Keywords:** high-contact sports, ice hockey injury, isolated manubrium fracture, sports-induced injury, surgical management of fractures

## Abstract

A 21-year-old, Division One men’s ice hockey athlete, sustained a traumatic, left shoulder injury that resulted in a displaced posterior left-sided manubrium fracture. Due to persistent pain that limited return to sport and fracture non-union, he underwent fracture fragment resection via an open transpectoralis major approach. The patient’s pain fully resolved, and he returned to sport-specific activities two months after surgery with the anticipation of full participation in contact activity upon return to campus. Isolated fractures to the manubrium, while uncommon, are generally associated with severe concomitant injuries. Contact sport athletes are at risk for manubrium fractures despite the few documented reports. Surgical management should be considered for cases of manubrium fractures in patients with persistent pain.

## Introduction

Isolated fractures to the manubrium account for a small subset of sternum fractures and are often caused by blunt chest trauma [1]. Documented reports of isolated manubrium fractures in athletes engaged in contact sports are limited, despite the risk of traumatic injury. Fractures to the manubrium are often managed non-operatively [2] and, in athletes, necessitate a prolonged return-to-sport protocol [3,4]. To our knowledge, there are no current reports of an isolated manubrium fracture managed surgically in an ice hockey athlete.

Here, we present a case of a non-union manubrium fracture in a collegiate ice hockey athlete and describe its surgical management, consisting of removal of the loose fragment via open exploration of the pectoralis muscle. The report discusses management of non-healing fractures in athletes as a means of functional recovery and pain management.

## Case presentation

Clinical history

**Figure 1:**
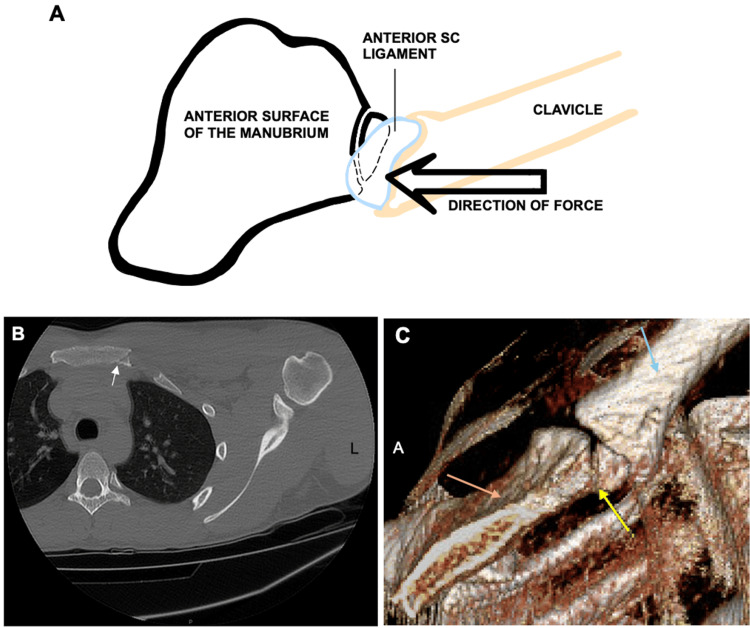
Representative mechanism of injury and computed tomography (CT) scan of the displaced fracture to the left posterior aspect of the manubrium. (A) Representative diagram of the anterior aspect of the manubrium indicating a fracture located on the upper left, posterior aspect. The arrow indicates the medially-directed force by the clavicle on the manubrium at the time of injury. (B) CT scan of the chest showing a minimally-displaced manubrium fracture in axial view (white arrow) post-injury. (C) 3D CT representation of a side view of the manubrium showing the anterior aspect of the left side of the manubrium (orange arrow) and demonstrating the posterior location of the fracture (yellow arrow) in proximity to the articular site of the left clavicle (blue arrow). Abbreviations: A = anterior; SC = sternoclavicular; L = left

A 21-year-old Division One men’s ice hockey athlete, with a remote history of a left clavicle fracture seven years prior, presented with left shoulder pain immediately after being checked into the boards during a collegiate game. On-site examination revealed pain with all active and passive range of motion (ROM) movements of the shoulder and tenderness to palpation over the distal left clavicle and sternoclavicular joint. The patient was immediately removed from the game and placed in a sling for comfort. Radiographs revealed no injury to the medial clavicle, but the manubrium was not well visualized. A computed tomography (CT) scan with intravenous contrast demonstrated a minimally displaced fracture of the upper left posterior manubrium, which extended into the sternoclavicular joint (Figure 1A-1C). The patient was initially treated with soft tissue mobilization, working on passive ROM. During the first week post-injury, the patient reported significant pain at rest and all activities of daily living, especially transitional movements (going from supine to sitting, rolling over in bed, and going from supine to standing). Seated lower extremity strengthening exercises were also initiated at this time.

At week three post-injury, the patient reported significant improvement in his pain at rest and during transitional movements. He tolerated passive and active ROM and started minimal sport-specific activities, including light skating and stick handling. Therapeutic approaches, including left shoulder isometrics, left wrist and elbow strengthening, and contralateral blood flow restriction therapy, were initiated. Given the atypical fracture, a bone health work-up was performed and revealed borderline low vitamin D levels. He completed a six-week, high-dose, Vitamin D course and was counseled on the importance of dietary calcium intake for fracture healing. By six weeks post-injury, the patient reported no pain during activities of daily living, rehabilitation exercises, weight room activity, or skating/stick handling, and minimal pain (1-2/10) with cross-body movement. The patient was subsequently cleared to start external rotation strengthening at a maximum of 20 pounds (as long as pain-free); however, without movement in the adduction or flexion planes. Additionally, at week eight, the patient was fitted with a bone stimulator in order to promote callus formation and bone healing.

By 10 weeks post-injury, pain with cross-body reaching and rolling activities increased. Furthermore, the patient reported episodic pain during left shoulder active ROM. Flexion adduction and internal rotation (FADIR), flexion abduction and external rotation (FABER), and push-up tests caused increased pain. Magnetic resonance imaging (MRI) was unremarkable for soft tissue damage and confirmed an unchanged posterior left-sided manubrium fracture that extended into the sternoclavicular joint with associated mild marrow edema. At this time, left shoulder strengthening exercises were stopped.

**Figure 2:**
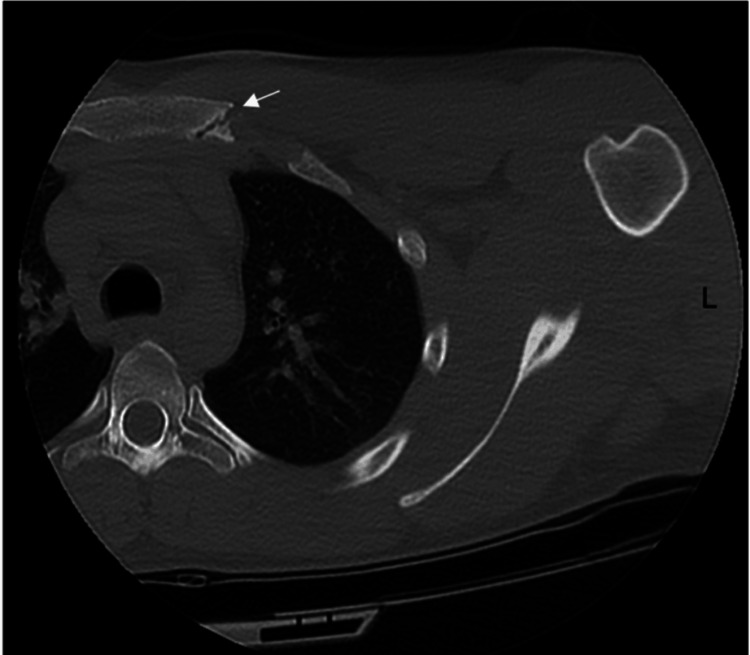
CT scan of the fractured manubrium three months post-injury. Axial view of the chest highlighting non-union of the left posterior aspect of the manubrium with minimal bony healing and sclerosis (white arrow).

A CT scan of the chest three months post-injury revealed minimal healing and persistence of the small triangular-shaped fragment off the left lateral and posterior aspect of the manubrium (Figure 2). It was advised that the athlete be allowed to return to upper extremity strengthening and hockey activities in a graded manner as tolerated. However, due to persistent pain, he was referred to a thoracic surgeon who reviewed the patient’s clinical course and fracture pattern and presented the option of removal of the loose manubrium fragment through open exploration via a transpectoralis major muscle approach. Given the location of the fracture fragment and the surgical approach, there was minimal concern for injury to underlying structures, including the mammary vessels. The patient opted for this surgical option due to his desire to return to hockey activities.

Surgical technique

The surgical procedure was performed 16 weeks post-injury. The patient was positioned supine on the operating table under general anesthesia. A hockey stick incision was made inferior to the left clavicle, extending to the lateral aspect of the manubrium and then vertically along the manubrium to access the site of the fracture. The pectoralis muscle was separated in the direction of its fibers, allowing access to the manubrium fracture without dividing the muscle itself. The manubrium fracture fragment was mobilized using cautery and a Cobb and was excised using a rongeur. Smaller and deeper fragments within the visual field were then removed, and the wound was subsequently irrigated. The left pleural space was entered during this procedure without injury to the underlying lung or adjacent structures.

Repalpation of the posterior aspect of the manubrium revealed complete excision of the fragment and no additional abnormalities. The pectoralis muscle was re-approximated using a running 2-0 Vicryl stitch to the deep layer and anterior layer. Closure of the skin was performed in standard fashion. The patient tolerated the procedure well with no postoperative complications. 

Postoperative follow-up

For the first 10 days following surgery, the patient engaged in minimal to no activity due to a small pneumothorax noted during surgery, which resolved within 24 hours. Initially, the patient was limited to passive ROM movements in all planes and progressed to active assisted ROM and shoulder isometrics in all planes by the end of week one of postoperative rehabilitation. Postoperative rehabilitation during weeks two through four included left upper extremity exercises using resistance bands in all planes. Toward the end of this period, the patient performed a push-up progression. The patient also began light skating and stick handling, which he tolerated well. A four-week postoperative radiograph revealed no acute cardiopulmonary processes or bony abnormalities. The patient was then progressed to pectoralis strengthening exercises using dumbbells limited to 5 lb, which progressed to 20 lb by the end of week six.

By two months post-surgery, the patient fully re-integrated into off-season training, including full weight training, conditioning, skating, and hockey skills with no limitations. The patient reports he has no functional limitations and has complete pain resolution. Given his recovery trajectory, he is deemed return-to-play ready. These results indicate an excellent outcome.

## Discussion

Fractures of the sternum account for less than one percent of sports-related upper extremity injuries [5]. Moreover, even fewer of these fractures are isolated to the manubrium and result in non-union. Notable cases include a 17-year-old and 20-year-old football players who sustained collisions while competing, causing sternum fractures, and a bodybuilder who suffered a sternum stress fracture due to excessive pectoral muscle stress [6-8]. There is also a report of an isolated distal sternum fracture in a female hockey athlete caused by a checking collision [9]. These cases were managed non-operatively and allowed for return to sport for these athletes within two to four months. While a large majority of fractures to the sternum, including manubrium fractures, are managed non-operatively, it is possible that surgical intervention might be necessary for optimal recovery and return to sport for elite athletes, as described for our patient.

Sternum fractures are especially concerning as the sternum is critical for protecting the underlying thoracic organs. These injuries are generally caused by falls, direct blunt trauma to the chest, or car accidents [1,10-12]. Sports-induced sternum fractures are generally less likely to occur because of lower force traumas to the chest and protective padding, but still pose a risk for associated complications.

Surgical treatment for these fractures is primarily limited to cases where obvious deformities exist [13]. Additionally, there is no standardized surgical approach for isolated manubrium fractures. This is likely due to the heterogeneity of fracture patterns and variability in grading the degree of bony deformity. Proposed surgical approaches are generally specific to a given fracture pattern. For example, Molina et al. proposed reduction and fixation of a posteriorly displaced manubrium fracture by exposing the entire length of the sternum through bilateral sub-thoracic incisions [14]. Bardos et al. describe fixation of vertical non-union manubrium fractures in elite athletes using partially threaded lag screws from the manubrium edges across the fracture site [4]. Our case involved fracture fragment removal via open exploration through a pectoralis muscle split. Radiographic images of the position and orientation of the fracture confirmed the feasibility of this approach; however, it may not be appropriate for other cases of manubrium fractures that have failed to heal.

Return-to-sport capacity and timeline are vital components of an athlete’s rehabilitation protocol. Generally, the progression of treatment of manubrium fractures can be measured by bony healing via imaging and functional tolerance in physical therapy. Pain levels are generally used to gauge injury severity and rehabilitation tolerance and can be monitored as a proxy for healing [15]. Pain can also be used as an indication for surgery; however, surgical decision-making primarily involves consideration of the location and type of fracture and the patient’s overall health. In our young athlete, surgical management was indicated by the patient’s worsening pain with active ROM movements and exercise, as well as radiographic evidence of fracture non-union. Previous efforts to manage his pain with physical therapy, while comprehensive, did not resolve his pain to a degree that would allow for a return to sport. In cases of manubrium fracture non-union in elite athletes, surgical management should be considered for improved outcomes and successful reintegration to sport.

## Conclusions

Here, we present an uncommon case of a non-union, isolated manubrium fracture in an ice hockey athlete that was caused by a checking collision during competition. The fracture was managed surgically and resulted in full functional recovery. The surgical procedure, which involved fracture fragment resection via an open trans-pectoralis major approach, was necessitated by the patient’s continuation of pain and non-healing fracture following an extensive rehabilitation protocol. Although uncommon, surgical management may be appropriate for manubrium fractures that have failed to heal, including cases without obvious structural deformities. Fracture pattern-specific approaches to managing manubrium fractures have been previously described; however, careful consideration of the injury type and patient goals is crucial for determining the appropriate surgical approach. This case report highlights the importance of case-dependent fracture management in order to achieve full return to sport for elite athletes.
